# Efficacy and safety of bevacizumab plus XELOX in patients with unresectable locally advanced or metastatic neuroendocrine tumors

**DOI:** 10.3389/fendo.2026.1939938

**Published:** 2026-09-30

**Authors:** Huike Wang, Huijing Tan, Yuting Sun, Yanwei Su, Yihebali Chi

**Affiliations:** Department of Medical Oncology, National Cancer Center/National Clinical Research Center for Cancer/Cancer Hospital, Chinese Academy of Medical Sciences and Peking Union Medical College, Beijing, China

**Keywords:** antiangiogenic therapy, bevacizumab, capecitabine, neuroendocrine tumors, oxaliplatin, retrospective study

## Abstract

Neuroendocrine tumors (NETs) are highly vascularized malignancies for which effective treatment options remain limited in the unresectable locally advanced or metastatic setting. Although platinum-based chemotherapy has demonstrated antitumor activity in advanced NETs, evidence supporting its combination with antiangiogenic therapy remains insufficient. In this retrospective study, 85 patients with progressive unresectable locally advanced or metastatic well-differentiated NETs initiated bevacizumab plus XELOX, consisting of bevacizumab at 7.5 mg/kg and oxaliplatin at 130 mg/m² every 3 weeks, together with capecitabine at 1,000 mg/m² twice daily on days 1–14 of each 21-day cycle. Among them, 72 patients who completed at least two treatment cycles and underwent post-baseline radiological evaluations comprised the safety set. The primary endpoint was objective response rate (ORR) by RECIST v1.1, while secondary endpoints included disease control rate (DCR), progression-free survival (PFS), overall survival (OS), and safety. Among the efficacy-evaluable patients, 62 (86.1%) received the regimen as second-line or later systemic therapy. Best responses included complete response (CR) in 1 patient (1.4%), partial response (PR) in 29 (40.3%), stable disease (SD) in 41 (56.9%), and progressive disease (PD) in 1 (1.4%), resulting in an ORR of 41.7% and a DCR of 98.6%. In a conservative sensitivity analysis encompassing all 85 treated patients—treating the 13 non-evaluable patients as non-responders—the ORR was 35.3%. After a median follow-up of 17.2 months, the median PFS was 20.9 months (95% CI, 13.7 months–not reached), with 6-, 12-, and 24-month PFS rates of 84.7%, 69.6%, and 47.3%, respectively. Median OS was not reached, and OS data remained immature. The most frequent any-grade adverse events (AEs) were elevated aspartate aminotransferase (48.6%), anemia (44.4%), lymphopenia and hypoalbuminemia (41.7% each), and hyperglycemia (40.3%), while neutropenia was the most frequent individual grade ≥3 AE (5.6%). No grade ≥3 bevacizumab-specific toxicities were observed. These findings suggest that bevacizumab plus XELOX demonstrates clinically meaningful antitumor activity and a manageable safety profile in advanced NET patients. Given the retrospective and single-arm design, this regimen may represent a potential alternative or sequential treatment option when alkylating-agent–based chemotherapy is unsuitable, whereas prospective comparative studies are warranted to validate these findings and determine its optimal clinical role.

## 1 Introduction

Neuroendocrine tumors (NETs) constitute a relatively rare yet highly heterogeneous group of malignancies originating primarily from the neuroendocrine system, with the gastrointestinal tract and lungs representing the most common primary sites (1, 2). Over the past five decades, the age-adjusted annual incidence of NETs has risen significantly (3). Despite their characteristically indolent progression, approximately 60%-80% of patients eventually develop distant metastases, rendering the management of advanced disease a considerable clinical challenge (1).

The management of neuroendocrine tumors has evolved into a multidisciplinary paradigm integrating diverse therapeutic modalities. Principal treatment options encompass somatostatin analogues (SSAs) for symptomatic control and tumor stabilization; molecularly targeted agents inhibiting specific signaling pathways, including mTOR inhibitors (everolimus) and multi-targeted tyrosine kinase inhibitors (sunitinib, surufatinib); cytotoxic chemotherapy (e.g., CAPTEM regimen—capecitabine plus temozolomide) for rapidly progressive disease (PD); peptide receptor radionuclide therapy (PRRT, e.g., ^177^Lu-DOTATATE) for tumors exhibiting high somatostatin receptor (SSTR) expression; and liver-directed interventions such as hepatic artery embolization and radiofrequency ablation for metastatic hepatic involvement (4–9). The absence of high-level evidence directly comparing these modalities and validated predictive biomarkers precludes the establishment of universal treatment sequencing (4, 5, 10). Consequently, therapeutic decisions necessitate comprehensive evaluation by multidisciplinary teams (MDT), incorporating primary tumor location, pathological grade, Ki-67 proliferation index, somatostatin receptor expression, tumor burden, and hepatic metastasis pattern.

Within this therapeutic landscape, chemotherapy maintains a significant position, particularly for aggressive NETs characterized by PD, high tumor burden, or elevated Ki-67 index (4, 5). Chemotherapeutic approaches assume heightened significance in pancreatic NETs (pNETs), where they typically demonstrate superior efficacy compared to intestinal counterparts. Alkylating agent-based regimens constitute the most frequently employed chemotherapeutic strategies, including streptozotocin (11, 12), dacarbazine (13), and temozolomide (14, 15), commonly administered in combination with fluoropyrimidines (5-fluorouracil or capecitabine).

Platinum-based chemotherapeutic agents demonstrate differential applicability across NETs subtypes. For aggressive variants, particularly poorly differentiated neuroendocrine carcinomas (NEC), cisplatin-etoposide combinations exhibit substantial efficacy and represent first-line standard therapy (16–18). Conversely, oxaliplatin-containing regimens (FOLFOX or XELOX/CAPEOX) have demonstrated activity across NETs spanning the differentiation spectrum (19–21). Notably, in well-differentiated pNETs, oxaliplatin-based chemotherapy has achieved impressive response rates, providing the fundamental rationale for the present investigation (20–22).

The pronounced vascularization of neuroendocrine tumors presents an additional therapeutic target, supported by documented overexpression of vascular endothelial growth factor (VEGF) and its receptors (VEGFRs) in both primary and metastatic lesions (23, 24). The clinical utility of antiangiogenic agents in advanced NET management is well established, exemplified by sunitinib in pNETs (25) and, more recently, surufatinib in both pNETs and extrapancreatic NETs (epNETs) (24, 26). Bevacizumab, a humanized monoclonal antibody targeting VEGF, has demonstrated antitumor activity as monotherapy in metastatic pNETs (27), with enhanced efficacy observed in combination with 5-fluorouracil-streptozotocin for pNETs (28) and superior outcomes when combined with capecitabine in gastrointestinal NETs (29). Nevertheless, evidence regarding the potential efficacy of bevacizumab combined with XELOX remains limited.

Therefore, leveraging the established efficacy of platinum-based chemotherapy in aggressive NETs, the superior tolerability profile of XELOX relative to cisplatin- or carboplatin-etoposide regimens, and the robust theoretical foundation supporting antiangiogenic therapy, this retrospective analysis aims to comprehensively evaluate the efficacy and safety of bevacizumab combined with XELOX in patients with rapidly progressive advanced NETs.

## 2 Materials and methods

### 2.1 Patient selection

This retrospective analysis enrolled patients with progressive advanced NETs who received at least one dose of bevacizumab plus XELOX at the National Cancer Center/National Clinical Research Center for Cancer/Cancer Hospital, Chinese Academy of Medical Sciences and Peking Union Medical College, from 2007 to 2026 (**Figure 1**). The inclusion criteria comprised: (1) histological or cytological confirmation of advanced progressive NETs; and (2) availability of complete clinical records. Patients who received at least one dose constituted the full analysis set (FAS; n = 85). The efficacy-evaluable set (EES) and safety set included patients who had received at least two treatment cycles and had at least one evaluable post-baseline tumor assessment (n = 72). Tumor classification and grading followed the 2019 World Health Organization (WHO) criteria, with well-differentiated NETs, including G1, G2, and G3, distinguished from poorly differentiated NECs.

**Figure 1:**
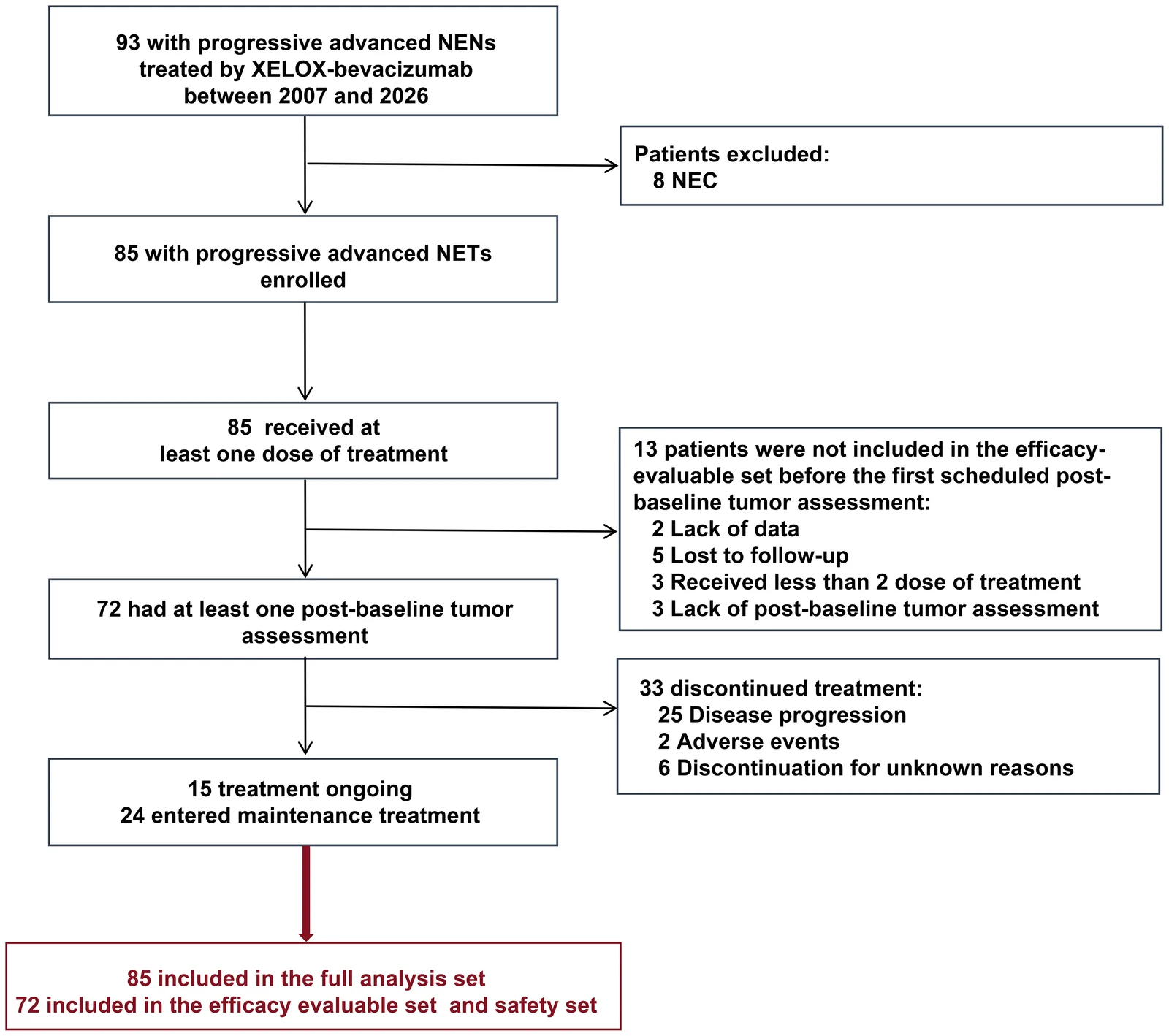
Flowchart of the study. NENs, neuroendocrine neoplasms; NEC, neuroendocrine carcinoma; NETs, neuroendocrine tumors.

### 2.2 Treatment strategy

Eligible patients received bevacizumab plus XELOX as induction chemotherapy in 21-day cycles until disease progression, unacceptable toxicity, patient refusal, or transition to maintenance therapy. Intravenous bevacizumab (7.5 mg/kg) and oxaliplatin (130 mg/m²) were administered on day 1, alongside oral capecitabine (1,000 mg/m² twice daily) from days 1 to 14, followed by a 7-day treatment hiatus. For patients who achieved sustained disease control and demonstrated good clinical tolerance after initial induction, maintenance therapy with bevacizumab plus capecitabine, capecitabine monotherapy, or bevacizumab monotherapy was permitted at the discretion of the treating oncologist. Maintenance regimens and individualized durations were physician-directed rather than protocol-mandated. Dosage adjustments were implemented according to primary tumor characteristics, metastatic burden, tumor dimensions, body surface area, hepatic and renal function, and bone marrow reserve at the discretion of the treating physicians.

Adverse events (AEs) were retrospectively collected from electronic medical records, nursing notes, and outpatient follow-up logs. All AEs were graded according to the National Cancer Institute Common Terminology Criteria for Adverse Events (CTCAE) version 4.0. To ensure a comprehensive safety profile, specific toxicities associated with bevacizumab (such as hypertension, proteinuria, bleeding events, and thromboembolism) and chemotherapy (such as peripheral neuropathy, diarrhea, and hand-foot syndrome) were systematically searched and extracted. Serious adverse events (SAEs), dose reductions, treatment interruptions, and permanent treatment discontinuations were documented.

### 2.3 Efficacy assessment

Baseline tumor assessments were performed by computed tomography (CT) or magnetic resonance imaging (MRI) within 4 weeks before treatment initiation. During the treatment phase, radiographic re-evaluations were performed at 6-week intervals. Tumor response was assessed retrospectively according to the Response Evaluation Criteria in Solid Tumors (RECIST) version 1.1 by at least two study investigators or radiologists independently, based on available serial CT or MRI scans. In cases of disagreement, the assessments were discussed to reach a consensus. Best overall response was recorded from treatment initiation until disease progression or treatment discontinuation. For patients achieving complete response (CR) or partial response (PR), confirmatory scans were evaluated when subsequent follow-up imaging was available in the medical records. The duration of response (DoR) was defined as the time from the first documented CR or PR to disease progression or death from any cause.

The primary endpoint was objective response rate (ORR), defined as the proportion of patients achieving CR or PR. Secondary endpoints comprised disease control rate (DCR, CR+PR+stable disease [SD]), progression-free survival (PFS was defined as the time from treatment initiation to radiographic disease progression or death from any cause, whichever occurred first), overall survival (OS was defined as the time from treatment initiation to death from any cause), and treatment safety. Survival status and subsequent antitumor therapies were documented via telephone follow-up. The final follow-up date was June 1, 2026.

### 2.4 Pathological and biomarker assessment

Pathological grading and tumor characteristics were assessed according to the applicable World Health Organization (WHO) classification. Immunohistochemical evaluation of MGMT, SSTR2, RB1, and PD-L1 was performed as part of routine clinical pathology in our institution. Protein expression levels of MGMT, SSTR2, and RB1 were categorized according to the scoring criteria recorded in clinical pathology records. PD-L1 expression was evaluated using the combined positive score (CPS). Molecular alterations were assessed using clinically available next-generation sequencing (NGS) testing of tumor specimens.

### 2.5 Statistical analysis

PFS and OS were estimated using the Kaplan–Meier method, and corresponding 95% confidence intervals (CIs) were calculated. Between-group survival distributions for exploratory subgroups were compared using the log-rank test. The median duration of follow-up was estimated using the reverse Kaplan–Meier method. All statistical analyses were exploratory in nature, and two-sided P values < 0.05 were considered statistically significant.

## 3 Results

### 3.1 Patient characteristics

The EES comprised 72 patients with progressive advanced NETs (**Table 1**). Forty-seven patients (65.3%) were male, and 39 (54.2%) were aged ≥60 years. The most common primary tumor sites were the pancreas in 26 patients (36.1%), colorectum in 14 (19.4%), mediastinum in 10 (13.9%), and lung in 9 (12.5%). Liver, bone, lung, and brain metastases were present in 51 (70.8%), 27 (37.5%), 17 (23.6%), and 2 patients (2.8%), respectively. Tumor grades were G1 in 6 patients (8.3%), G2 in 46 (63.9%), and G3 in 20 (27.8%). 46 patients (63.9%) received ≥8 cycles of bevacizumab plus XELOX. The regimen was administered as second-line or later systemic therapy in 62 patients (86.1%) and as third-line or later therapy in 51 (70.8%). 31 patients (43.1%) had undergone surgery (**Supplementary Table 1**).

**Table 1:** Baseline demographic and clinical characteristics of the study cohort (N = 72).

Characteristics	All patients N= 72 (%)
Sex	
Female	25 (34.7)
Male	47 (65.3)
Age, years	
<60	33 (45.8)
≥60	39 (54.2)
Family history	
No	64 (88.9)
Yes	8 (11.1)
Smoking	
No	60 (83.3)
Yes	12 (16.7)
Drinking	
No	63 (87.5)
Yes	9 (12.5)
Hypertension	
No	59 (81.9)
Yes	13 (18.1)
Diabetes	
No	62 (86.1)
Yes	10 (13.9)
Carcinoid syndrome	
No	69 (95.8)
Yes	3 (4.2)
Primary tumour site	
Pancreas	26 (36.1)
Colorectum	14 (19.4)
Small intestine	3 (4.2)
Mediastinum	10 (13.9)
Lung	9 (12.5)
Liver	3 (4.2)
Stomach	2 (2.8)
Others	5 (6.9)
Primary tumour site	
Pancreas	26 (36.1)
Non-pancreas	46 (63.9)
Primary tumour site	
Lung	9 (12.5)
Non-lung	63 (87.5)
Radiotherapy	
No	59 (81.9)
Yes	13 (18.1)
Interventional therapy	
No	44 (61.1)
Yes	28 (38.9)
Immunotherapy	
No	63 (87.5)
Yes	9 (12.5)
Neoadjuvant therapy	
No	67 (93.1)
Yes	5 (6.9)
Surgery	
No	41 (56.9)
Yes	31 (43.1)
Number of lines of systemic treatment	
1	10 (13.9)
2	11 (15.3)
3	17 (23.6)
4	20 (27.8)
≥5	14 (19.4)
Number of treatment cycles	
<8	26 (36.1)
≥8	46 (63.9)
Number of metastatic organs	
No distant metastasis	4 (5.6)
≤2 organs	50 (69.4)
≥3 organs	18 (25.0)
Bone metastasis	
No	45 (62.5)
Yes	27 (37.5)
Brain metastasis	
No	70 (97.2)
Yes	2 (2.8)
Liver metastasis	
No	21 (29.2)
Yes	51 (70.8)
Lung metastasis	
No	55 (76.4)
Yes	17 (23.6)
Tumor size, cm	
≤3.5	34 (47.2)
>3.5	38 (52.8)
Grade	
G1	6 (8.3)
G2	46 (63.9)
G3	20 (27.8)
AJCC T staging	
T1	20 (27.8)
T2	30 (41.7)
T3	18 (25.0)
T4	4 (5.6)
AJCC N staging	
N0	19 (26.4)
N1	42 (58.3)
N2	11 (15.3)
AJCC M staging	
M0	4 (5.6)
M1	68 (94.4)
Vascular invasion	
No	18 (25.0)
Yes	15 (20.8)
Unknown	39 (54.2)
Nerve invasion	
No	23 (31.9)
Yes	10 (13.9)
Unknown	39 (54.2)
Ki-67 level	
<5%	5 (6.9)
5−10%	23 (31.9)
>10%	44 (61.1)
MGMT status	
MGMT 0 or 1+	23 (31.9)
MGMT 2+ or 3+	39 (54.2)
Unknown	10 (13.9)
SSTR2 status	
SSTR2 0 or 1+	29 (40.3)
SSTR2 2+ or 3+	37 (51.4)
Unknown	6 (8.3)
RB1 status	
RB1 0 or 1+	48 (66.7)
RB1 2+or 3+	11 (15.3)
Unknown	13 (18.1)
PD-L1 CPS	
0	45 (62.5)
≥1	16 (22.2)
unknown	11 (15.3)
MSS/MSI	
MSS	57 (79.2)
MSI	2 (2.8)
unknown	13 (18.1)
Gene mutation	
No	33 (45.8)
Yes	13 (18.1)
unknown	26 (36.1)
Ca19-9 (U/mL)	
≤ 20.53	41 (56.9)
> 20.53	31 (43.1)
Ca72-4 (U/mL)	
≤ 2.47	44 (61.1)
> 2.47	28 (38.9)
AFP (ng/mL)	
≤ 4.09	41 (56.9)
> 4.09	31 (43.1)
NSE (ng/mL)	
≤ 20.54	39 (54.2)
> 20.54	33 (45.8)
CEA (ng/mL)	
≤ 2.70	37 (51.4)
> 2.70	35 (48.6)
ProGRP (ng/mL)	
≤ 287.72	53 (73.6)
> 287.72	19 (26.4)

### 3.2 Treatment efficacy

In EES patients received a median of 8 cycles (range 2–25) of bevacizumab plus XELOX. Treatment duration exceeded 6 months in 47 patients (65.3%). According to RECIST 1.1, the ORR was 41.7% (30/72; 95% CI, 30.2%–53.9%), and the DCR was 98.6% (71/72; 95% CI, 92.5%–100.0%). In a conservative sensitivity analysis encompassing the entire full analysis set (n = 85), treating the 13 non-evaluable patients as non-responders, the ORR was 35.3%.

Subgroup analyses revealed numerically higher ORR in pNETs (53.8% vs 34.8% in epNETs, *P* = 0.115), MGMT expression 0/1+ (47.8% vs 38.5% in MGMT 2+ or 3+, *P* = 0.765), and G1 grade (50.0% vs 41.3% in G2 and 40.0% in G3, P = 0.906), though these differences were not statistically significant (**Table 2**). The waterfall chart (**Figure 2**) depicts best overall responses: CR in 1 (1.4%), PR in 29 (40.3%), SD in 41 (56.9%), and PD in 1 (1.4%). Median time to best imaging response was 1.7 months (**Figure 3**), 15 patients (20.8%) continued treatment, 24 (33.3%) initiated maintenance therapy, 25 (34.7%) experienced progression and switched regimens, 6 (8.3%) discontinued treatment, and 2 (2.8%) switched regimens due to AEs. Temporal changes in target lesion dimensions are depicted in **Figure 4**.

**Table 2:** Objective response rates with 95% confidence intervals according to primary tumor site and tumor grade.

Subgroup	Evaluated (n)	Responders (n, CR+PR)	ORR (%)	95% CI (%)	P value
Primary tumour site					0.077
Pancreas	26	14	53.8	33.4-73.4	
Colorectum	14	4	28.6	8.4-58.1	
Small intestine	3	1	33.3	0.8-90.6	
Mediastinum	10	6	60.0	26.2-87.8	
Lung	9	3	33.3	7.5-70.1	
Stomach	2	2	100.0	15.8-100.0	
Others	8	0	0.0	0.0-36.9	
Primary tumour site					0.115
Pancreas	26	14	53.8	33.4-73.4	
Non-pancreas	46	16	34.8	21.4-50.2	
Grade					0.906
G1	6	3	50.0	11.8-88.2	
G2	46	19	41.3	27.0-56.8	
G3	20	8	40.0	19.1-63.9	

**Figure 2:**
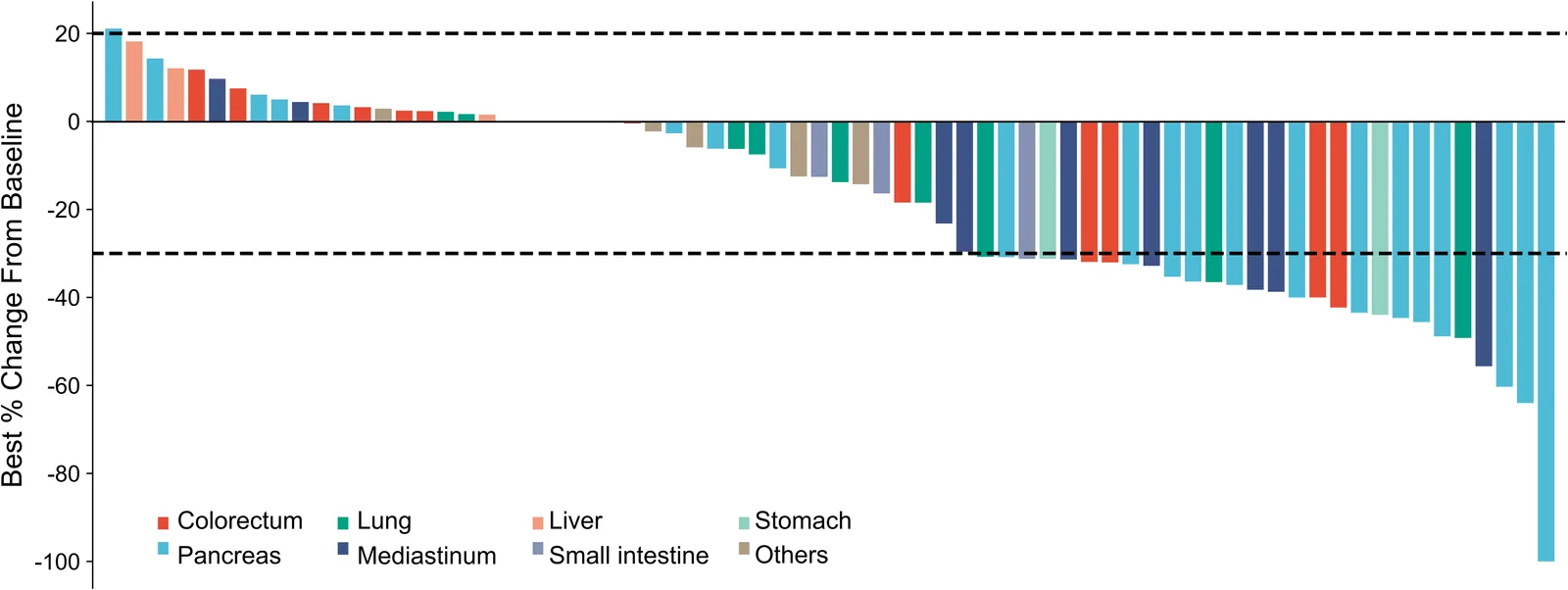
Waterfall plot of best tumor response according to RECIST version 1.1.

**Figure 3:**
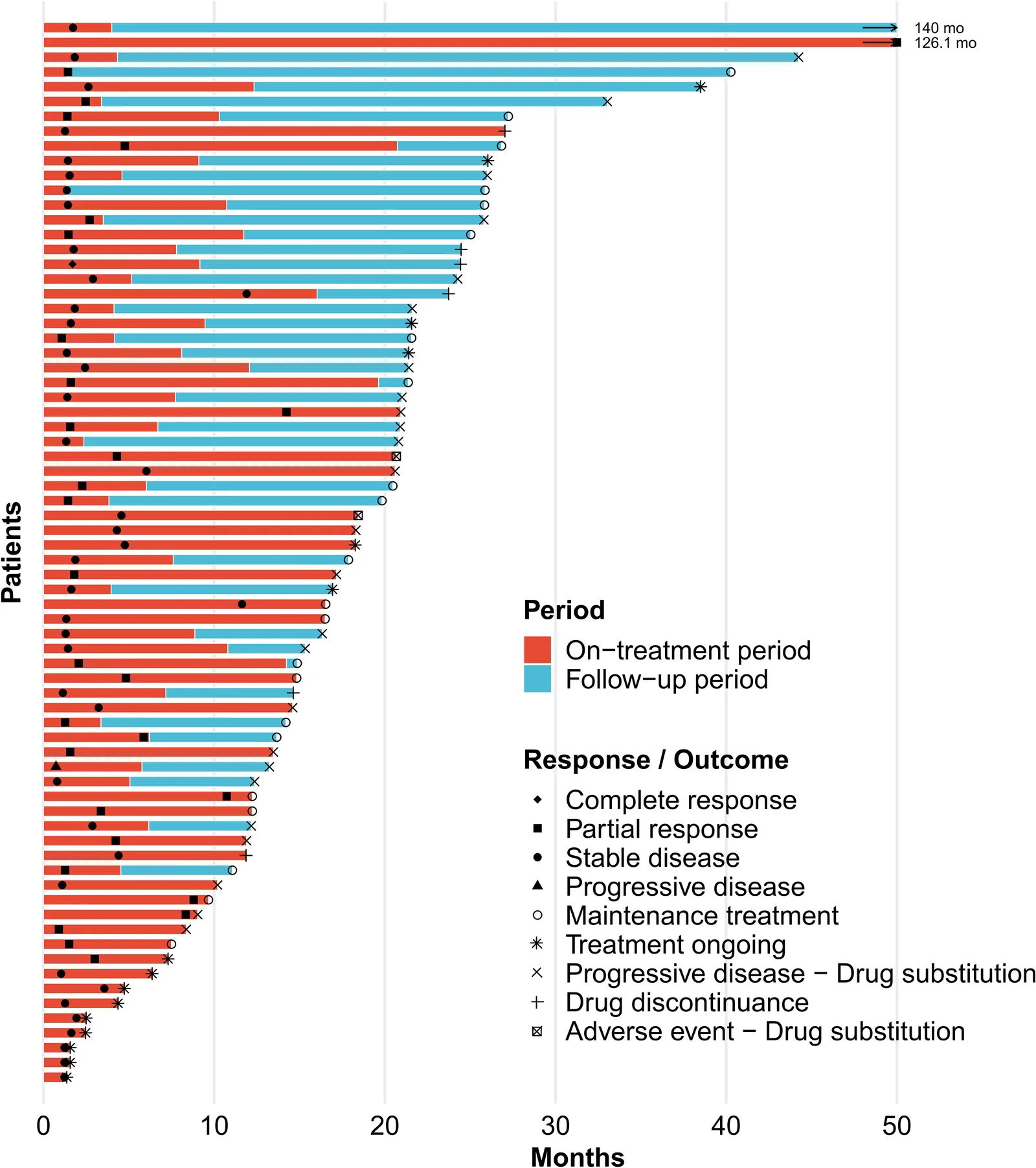
Swimmer plot showing the type and duration of tumor response in evaluable patients.

**Figure 4:**
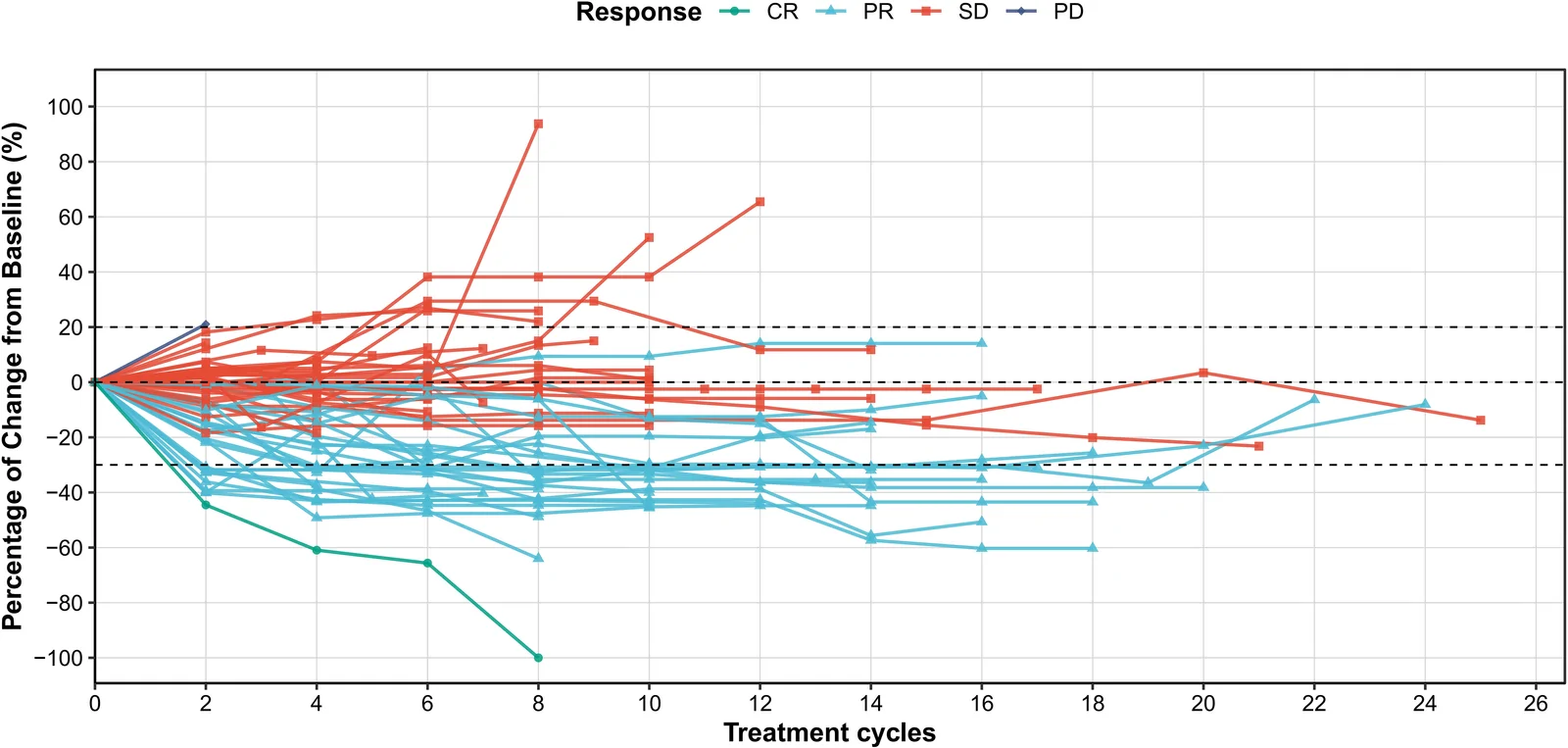
Spider plots showing longitudinal percentage changes in target-lesion diameter from baseline. Downward trajectories indicate tumor shrinkage, whereas upward trajectories indicate tumor growth. PR, partial response; SD, stable disease; CR, complete response; PD, progressive disease.

### 3.3 Survival analysis

At the data cutoff, 27 PFS events and 5 deaths had occurred. The median follow-up duration was 17.2 months (95% CI, 14.6–20.6 months). Median PFS was 20.9 months (95% CI, 13.7 months–not reached). The estimated PFS rates at 6, 12, and 24 months were 84.7% (95% CI, 73.5%–91.5%), 69.6% (95% CI, 56.4%–79.5%), and 47.3% (95% CI, 29.8%–63.0%), respectively. Median OS was not reached. The estimated OS rates at 12, 24, and 36 months were 93.5% (95% CI, 83.6%–97.5%), 93.5% (95% CI, 83.6%–97.5%), and 83.1% (95% CI, 50.8%–95.1%), respectively (**Figure 5**). Subgroup survival analyses demonstrated significantly inferior OS in patients with carcinoid syndrome (*P* < 0.001), or lung metastases (*P* = 0.046), and inferior PFS in those with prior immunotherapy (*P* = 0.030). No other variables showed significant associations in subgroup analysis (**Supplementary Figures 1**, **2**).

**Figure 5:**
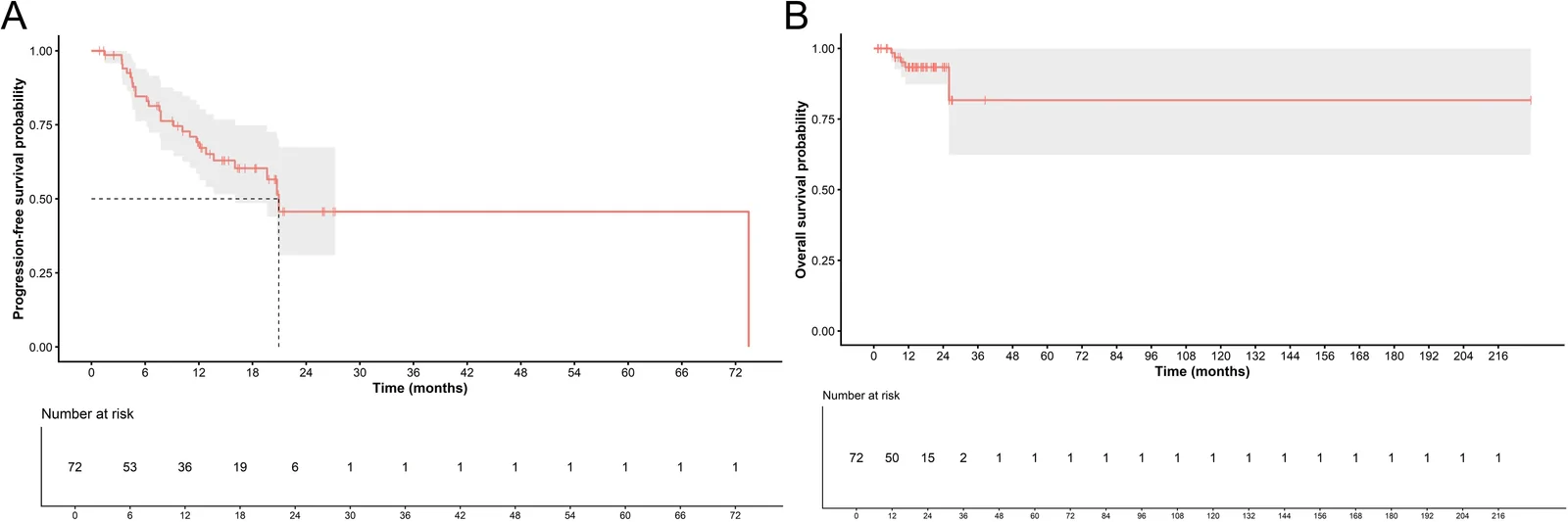
Kaplan–Meier estimates of **(A)** progression-free survival and **(B)** overall survival.

### 3.4 Treatment safety

All 72 patients were evaluable for safety (**Table 3**). Treatment-related AEs of any grade occurred in 55 patients (76.4%), and grade ≥ 3 AEs occurred in 17 patients (23.6%). Serious adverse events (SAEs) occurred in 4 patients (5.6%). The most frequent hematological and biochemical toxicities included elevated aspartate aminotransferase (48.6%), anemia (44.4%), lymphopenia (41.7%), hypoalbuminemia (41.7%), and hyperglycemia (40.3%).Regarding targeted agent-associated toxicities of particular interest, hypertension of any grade was observed in 11 patients (15.3%, all grade 1–2), and proteinuria of any grade occurred in 14 patients (19.4%, all grade 1–2; **Table 3**). No grade ≥ 3 hypertension, proteinuria, severe bleeding events, or arterial/venous thromboembolism were observed in this cohort. Peripheral neuropathy and hand-foot syndrome were manageable and predominantly grade 1–2. Dose modifications included: oxaliplatin dose reduction in 2 patients and discontinuation in 1 due to intolerance; capecitabine dose reduction in 1 patient; concurrent reduction of both chemotherapeutic agents in 1 patient; and bevacizumab discontinuation in 1 patient. Three patients permanently discontinued treatment due to AEs, primarily jaundice and hand-foot syndrome.

**Table 3:** Treatment-emergent adverse events among 72 patients.

Characteristics	Any grade n (%)	G1 n (%)	G2 n (%)	G3 n (%)	G4 n (%)
Hematological toxicities					
Anemia	32 (44.45)	22 (30.56)	9 (12.50)	1 (1.39)	0 (0.00)
Lymphopenia	30 (41.67)	15 (20.83)	12 (16.67)	2 (2.78)	1 (1.39)
Neutropenia	20 (27.78)	6 (8.33)	10 (13.89)	4 (5.56)	0 (0.00)
Thrombocytopenia	20 (27.78)	8 (11.11)	11 (15.28)	0 (0.00)	1 (1.39)
Leukopenia	14 (19.44)	5 (6.94)	7 (9.72)	2 (2.78)	0 (0.00)
Targeted/chemotherapy-specific toxicities					
Elevated aspartate aminotransferase	35 (48.61)	28 (38.89)	4 (5.56)	3 (4.17)	0 (0.00)
Hyperglycemia	29 (40.28)	25 (34.72)	4 (5.56)	0 (0.00)	0 (0.00)
Hypoalbuminemia	30 (41.67)	28 (38.89)	2 (2.78)	0 (0.00)	0 (0.00)
Elevated alkaline phosphatase	25 (34.72)	20 (27.78)	4 (5.56)	1 (1.39)	0 (0.00)
Elevated alanine aminotransferase	14 (19.44)	11 (15.28)	1 (1.39)	2 (2.78)	0 (0.00)
Proteinuria	14 (19.44)	12 (16.67)	2 (2.78)	0 (0.00)	0 (0.00)
Hyperbilirubinemia	14 (19.44)	10 (13.89)	4 (5.56)	0 (0.00)	0 (0.00)
Hypokalemia	8 (11.11)	5 (6.94)	3 (4.17)	0 (0.00)	0 (0.00)
Hypercholesterolemia	5 (6.94)	3 (4.17)	2 (2.78)	0 (0.00)	0 (0.00)
Bleeding events	3 (4.17)	2 (2.78)	1 (1.39)	0 (0.00)	0 (0.00)
Peripheral neuropathy	15 (20.83)	10 (13.89)	5 (6.94)	0 (0.00)	0 (0.00)
Hand-foot syndrome	12 (16.67)	7 (9.72)	4 (5.56)	1 (1.39)	0 (0.00)
Diarrhea	10 (13.89)	6 (8.33)	4 (5.56)	0 (0.00)	0 (0.00)
Treatment modification/disposition summary					
Serious adverse events (SAEs)	4 (5.56)	—	—	—	—
Dose reduction/interruption due to AEs	5 (6.94)	—	—	—	—
Permanent treatment discontinuation due to AEs	3 (4.17)	—	—	—	—
Treatment-related deaths	0 (0.00)	—	—	—	—

### 3.5 Genetic mutations

Genetic-testing status was available for 46 patients: 13 were mutation-positive and 33 had no detected alteration, corresponding to 28.3% of tested patients and 18.1% of the full cohort. Detailed variant-level information was available for 13 cases in **Figure 6** and **Table 4**. Across these cases, 24 variants were listed: 9 pathogenic variants, 10 likely pathogenic variants, and 5 variants of uncertain significance. Recurrently listed genes included MEN1, TP53, TSC2, and SDHA. The most frequently mutated genes were MEN1, TP53, and TSC2. Somatic sequencing in two patients with primary pNETs identified co-occurring TP53 and TSC2 variants. Case D11 had a TSC2 exon 40 variant (c.5121_5123delinsA, p.N1707Kfs*21; variant allele frequency, 9.1%) and a TP53 exon 8 variant (c.825T>G, p.C275W; 16.2%). Case D12 had a TSC2 exon 35 frameshift variant (p.P1508fs; 22.8%) and a TP53 exon 5 variant (p.R181C; 18.6%). Both patients achieved PR. Case D8 carried a germline MEN1 alteration and achieved SD, whereas Case D12 also harbored a somatic MEN1 exon 2 frameshift variant (p.A49fs; 39.6%) and achieved PR.

**Figure 6:**
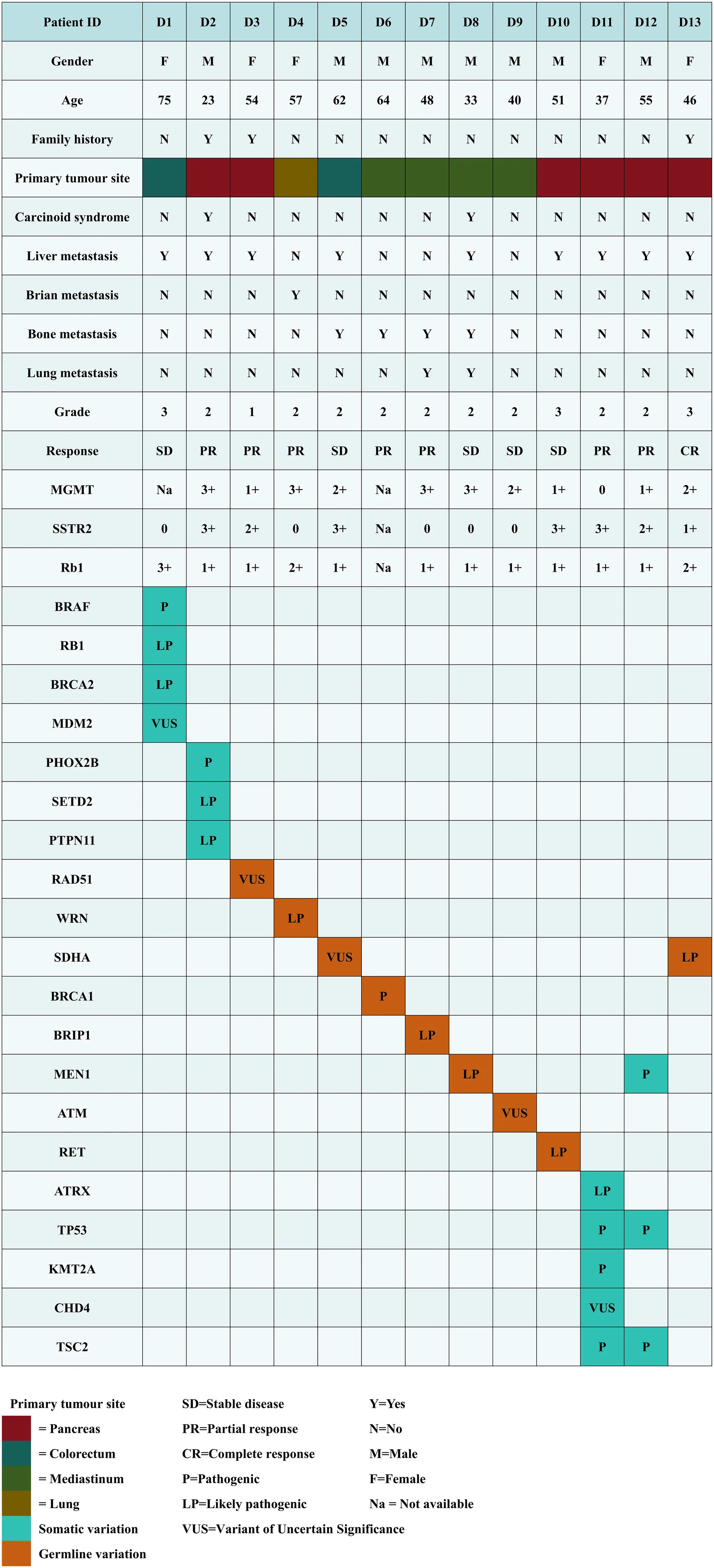
Clinical and molecular characterization of 13 cases with detailed genetic alterations.

**Table 4:** Somatic and germline variants identified in 13 patients with neuroendocrine tumors.

Genes	Mutation type	Variant type	cDNA mutation	Protein alteration	Variant allele frequency %	Variant classification
*BRAF*	Somatic mutation	Mutation of Exon 15	c.1799T>A	p.V600E	27.1%	Pathogenic
*RB1*	Somatic mutation	Mutation of Exon 18	—	p.T583I	11.2%	Likely pathogenic
*BRCA2*	Somatic mutation	Mutation of Exon 21	—	p.T3033fs	5.1%	Likely pathogenic
*MDM2*	Somatic mutation	Mutation of Exon 4	—	p.R71*	11.5%	Uncertain significance
*PHOX2B*	Somatic mutation	Mutation of Exon 3	c.738_776del	p.A248_A260del	20.2%	Pathogenic
*SETD2*	Somatic mutation	SETD2-intergenic(ERICH4,PCAT19)	—	—	13.7%	Likely pathogenic
*PTPN11*	Somatic mutation	intergenic(SPATA31D1,RASEF)-PTPN11	—	—	29.5%	Likely pathogenic
*MEN1*	Somatic mutation	Mutation of Exon 2	—	p.A49fs	39.6%	Pathogenic
*ATRX*	Somatic mutation	Mutation of Exon 30	c.6589C>T	p.R2197C	23.4%	Likely pathogenic
*TP53*	Somatic mutation	Mutation of Exon 8	c.825T>G	p.C275W	16.2%	Pathogenic
*TP53*	Somatic mutation	Mutation of Exon 5	—	p.R181C	18.6%	Pathogenic
*KMT2A*	Somatic mutation	Mutation of Exon 20	c.5623C>T	p.Q1875*	14.6%	Pathogenic
*CHD4*	Somatic mutation	Mutation of Exon 17	c.263C>G	p.A868G	12.8%	Uncertain significance
*TSC2*	Somatic mutation	Mutation of Exon 40	c.5121_5123delinsA	p.N1707Kfs*21	9.1%	Pathogenic
*TSC2*	Somatic mutation	Mutation of Exon 35	—	p.P1508fs	22.8%	Pathogenic
*RAD51*	Germline mutation	NM_00164269.1 heterozygous mutation	c.1dup	p.Met1Asnfs*8	—	Uncertain significance
*WRN*	Germline mutation	NM_000553.4 heterozygous mutation	c.2517C>G	p.Tyr839*	—	Likely pathogenic
*SDHA*	Germline mutation	NM_004168.3 heterozygous mutation	c.991G>A	p.Ala331Thr	—	Uncertain significance
*SDHA*	Germline mutation	NM_004168.3 heterozygous mutation	c.1534C>T	p.Arg512*	—	Likely pathogenic
*BRCA1*	Germline mutation	NM_007294.3 heterozygous mutation	c.5470_5477del	p.Ile1824fs	—	Pathogenic
*BRIP1*	Germline mutation	NM_032043.2 heterozygous mutation	c.1A>G	p.Met1?	—	Likely pathogenic
*MEN1*	Germline mutation	NM_000244.3 heterozygous mutation	—	—	—	Likely pathogenic
*RET*	Germline mutation	NM_000051.3 heterozygous mutation	c.2410G>T	p.Val804Leu	—	Likely pathogenic
*ATM*	Germline mutation	NM_000051.3 heterozygous mutation	c.1037T>A	p.Ile346Asn	—	Uncertain significance

## 4 Discussion

This retrospective study included 72 patients with unresectable locally advanced or metastatic NETs as the efficacy evaluable set to evaluate the efficacy and safety of bevacizumab plus XELOX. The cohort was heavily pretreated, with 86.1% of patients receiving the regimen as second-line or later therapy and 70.8% as third-line or later therapy. The regimen achieved an ORR of 41.7% and a DCR of 98.6%. The safety profile was manageable, with predominantly grade 1–2 treatment-related toxicities. These findings provide additional clinical support for the chemotherapy plus antiangiogenic strategy in progressive advanced NETs.

To contextualize the clinical positioning of bevacizumab plus XELOX, we performed comparative analyses with contemporary standard therapies for advanced NETs. Chemotherapy-antiangiogenic combinations represent an important therapeutic direction in advanced NETs. Bevacizumab-based strategies have demonstrated clinically meaningful antitumor activity in several studies (28–33). Previous investigations reported ORR of 42%-56% for bevacizumab combined with various chemotherapeutic regimens in advanced pNETs (28, 29), contrasting with conventional FOLFOX (ORR 10%-29%) and bevacizumab monotherapy (ORR 13%) (20, 21, 34–39). The BETTER trial established bevacizumab plus capecitabine as a therapeutic option in 49 treatment-naive patients with gastrointestinal NETs, demonstrating an 18% ORR and median PFS of 23.4 months (29). Although the antitumor activity observed with bevacizumab plus XELOX compares favorably with historical outcomes reported for conventional chemotherapy or antiangiogenic monotherapy in advanced NETs, direct cross-trial comparisons should be interpreted with caution due to confounding factors, including differing patient selection, prior treatment lines, baseline tumor burden, and heterogeneous anatomical origins.

CAPTEM (capecitabine plus temozolomide) is an established alkylating-agent regimen for NETs, particularly pNETs. A retrospective analysis by Al-Toubah et al. involving 462 patients reported an ORR of 51.5% and a median PFS of 23 months with CAPTEM (40). The efficacy of CAPTEM may be associated with biomarker status, particularly O^6^-methylguanine-DNA methyltransferase (MGMT) expression, and its use may also be limited by alkylating agent–related toxicity. Several studies have associated low MGMT protein expression with greater sensitivity to alkylating agents, with reported ORRs of approximately 55%–60% in tumors with low MGMT expression compared with 10%–20% in those with high expression (41–43). In contrast, the activity of platinum-based chemotherapy appears to be less dependent on MGMT expression, suggesting that platinum-containing regimens may retain activity across different MGMT-expression categories (43). In the present cohort, bevacizumab plus XELOX achieved an overall ORR of 41.7%. Among patients with available MGMT results, the ORR was 47.8% in the MGMT 0/1+ group and 38.5% in the MGMT 2+/3+ group, with no statistically significant difference between the groups. Although the absence of a statistically significant difference does not establish that MGMT is irrelevant to treatment selection, these exploratory findings suggest that the activity of bevacizumab plus XELOX may be less dependent on MGMT status. This indicates that the regimen may provide a potential non-alkylating treatment option for patients with high MGMT expression or for those who are intolerant to alkylating agents. However, given the retrospective design and limited sample size, prospective validation is required.

Notably, an ORR of 40.0% was observed in patients with G3 NETs within our cohort, aligning with evolving treatment paradigms for this subset. This finding is contextualized by the multicenter analysis from Apostolidis et al., which reported ORRs of 56% for FOLFOX versus 27% for CAPTEM in G3 NETs (44). These findings provide a rationale for further prospective evaluation of oxaliplatin-based chemotherapy in this subgroup.

Furthermore, our exploratory genetic profiling revealed that 2 pNET cases with detailed sequencing data and co-occurring TP53 and TSC2 variants achieved PR. TP53 alterations are frequently linked to genomic instability and aggressive tumor behavior, while TSC2 acts as a key negative regulator of the mTOR pathway, and its inactivation is postulated to drive upregulation of the VEGF/VEGFR signaling axis (45, 46). The co-occurrence of TP53 and TSC2 variants in two responding patients was hypothesis-generating. This extremely small subset precludes any definitive assessment of predictive value, and this observation remains strictly hypothesis-generating.

The observed toxicity pattern was generally consistent with the known safety profiles of bevacizumab, oxaliplatin, and capecitabine. Most treatment-related adverse events were grade 1–2, whereas grade 3–4 events, including neutropenia and elevated transaminases, were relatively uncommon. However, because adverse events were collected retrospectively, low-grade or outpatient toxicities may have been incompletely documented. Notably, after achieving disease control with the initial combination regimen, a subset of patients transitioned to maintenance therapy with bevacizumab and/or capecitabine. This induction–maintenance approach may help preserve antitumor activity while reducing cumulative treatment burden and toxicity, and therefore represents a potentially practical strategy in clinical management. Nevertheless, because maintenance regimens were not standardized and no control group was available, its contribution to efficacy and safety requires confirmation in prospective studies.

Several limitations should be acknowledged. First, this single-center retrospective study lacked a contemporaneous control group and included only patients who completed at least two treatment cycles, potentially introducing selection and survivorship bias. Second, the inclusion of NETs from multiple primary sites, including pulmonary and mediastinal tumors, reflects real-world clinical practice but also introduces biological and treatment heterogeneity that limits site-specific interpretation. Third, radiographic assessment was not centrally reviewed, biomarker data were incomplete, and retrospective AE collection may have underestimated toxicity. In addition, the limited number of PFS events and deaths reduced the statistical power of survival analyses and increased the risk of unstable or false-positive exploratory findings. Finally, the observed TP53 and TSC2 co-mutations were based on very few cases and should be considered hypothesis-generating.

In conclusion, bevacizumab plus XELOX demonstrated antitumor activity and a generally manageable safety profile in patients with progressive advanced NETs, including those who had received multiple prior lines of therapy. The regimen may provide a feasible alternative or sequential treatment option when alkylating-agent–based chemotherapy is unsuitable, ineffective, or poorly tolerated. Nevertheless, these findings should be interpreted cautiously given the retrospective design and limited survival events. Prospective studies are needed to confirm the clinical role of this regimen and to explore whether biomarkers such as MGMT expression or selected molecular alterations may have predictive relevance.

## Data Availability

The raw data supporting the conclusions of this article will be made available by the authors, without undue reservation.

## References

[1] Abuzakhm SM Sukrithan V Fruth B Qin R Strosberg J Hobday TJ . A phase II study of bevacizumab and temsirolimus in advanced extra-pancreatic neuroendocrine tumors. Endocr Relat Cancer. (2023) 30:e220301. doi: 10.1530/ERC-22-0301 37702588 PMC10585708

[2] Al-Toubah T Morse B Pelle E Strosberg J . Efficacy of FOLFOX in patients with aggressive pancreatic neuroendocrine tumors after prior capecitabine/temozolomide. Oncologist. (2021) 26:115–9. doi: 10.1002/onco.13611 33225478 PMC7873336

[3] Al-Toubah T Pelle E Valone T Haider M Strosberg JR . Efficacy and toxicity analysis of capecitabine and temozolomide in neuroendocrine neoplasms. J Natl Compr Canc Netw. (2021) 20:29–36. doi: 10.6004/jnccn.2021.7017 34433130

[4] Apostolidis L Dal Buono A Merola E Jann H Jäger D Wiedenmann B . Multicenter analysis of treatment outcomes for systemic therapy in well-differentiated grade 3 neuroendocrine tumors (NET G3). Cancers (Basel). (2021) 13:1936. doi: 10.3390/cancers13081936 33923759 PMC8073753

[5] Basu B Sirohi B Corrie P . Systemic therapy for neuroendocrine tumours of gastroenteropancreatic origin. Endocr Relat Cancer. (2010) 17:R75–90. doi: 10.1677/ERC-09-0108 20008097

[6] Berruti A Fazio N Ferrero A Brizzi MP Volante M Nobili E . Bevacizumab plus octreotide and metronomic capecitabine in patients with metastatic well-to-moderately differentiated neuroendocrine tumors: the XELBEVOCT study. BMC Cancer. (2014) 14:184. doi: 10.1186/1471-2407-14-184 24628963 PMC3996907

[7] Castellano D Capdevila J Sastre J Alonso V Llanos M García-Carbonero R . Sorafenib and bevacizumab combination targeted therapy in advanced neuroendocrine tumour: a phase II study of the Spanish Neuroendocrine Tumour Group (GETNE0801). Eur J Cancer. (2013) 49:3780–7. doi: 10.1016/j.ejca.2013.06.042 24012098

[8] Chan JA Stuart K Earle CC Clark JW Bhargava P Miksad R . Prospective study of bevacizumab plus temozolomide in patients with advanced neuroendocrine tumors. J Clin Oncol. (2012) 30:2963–8. doi: 10.1200/JCO.2011.40.3147 22778320 PMC4874232

[9] Clewemar Antonodimitrakis P Sundin A Wassberg C Granberg D Skogseid B Eriksson B . Streptozocin and 5-fluorouracil for the treatment of pancreatic neuroendocrine tumors: efficacy, prognostic factors, and toxicity. Neuroendocrinology. (2016) 103:345–53. doi: 10.1159/000439086 26279284

[10] Couvelard A O'Toole D Turley H Leek R Sauvanet A Degott C . Microvascular density and hypoxia-inducible factor pathway in pancreatic endocrine tumours: negative correlation of microvascular density and VEGF expression with tumour progression. Br J Cancer. (2005) 92:94–101. doi: 10.1038/sj.bjc.6602245 15558070 PMC2361752

[11] Cros J Hentic O Rebours V Zappa M Gille N Theou-Anton N . MGMT expression predicts response to temozolomide in pancreatic neuroendocrine tumors. Endocr Relat Cancer. (2016) 23:625–33. doi: 10.1530/ERC-16-0117 27353036

[12] Dasari A Shen C Halperin D Zhao B Zhou S Xu Y . Trends in the incidence, prevalence, and survival outcomes in patients with neuroendocrine tumors in the United States. JAMA Oncol. (2017) 3:1335–42. doi: 10.1001/jamaoncol.2017.0589 28448665 PMC5824320

[13] de Mestier L Couvelard A Blazevic A Hentic O de Herder WW Rebours V . Critical appraisal of MGMT in digestive NET treated with alkylating agents. Endocr Relat Cancer. (2020) 27:R391–405. doi: 10.1530/ERC-20-0227 32698158

[14] de Mestier L Lepage C Baudin E Coriat R Courbon F Couvelard A . Digestive neuroendocrine neoplasms: French intergroup clinical practice guidelines for diagnosis, treatment, and follow-up. Dig Liver Dis. (2020) 52:473–92. doi: 10.1016/j.dld.2020.02.011 32234416

[15] de Mestier L Walter T Brixi H Evrard C Legoux JL de Boissieu P . Comparison of temozolomide-capecitabine to 5-fluorouracil-dacarbazine in 247 patients with advanced digestive neuroendocrine tumors using propensity score analyses. Neuroendocrinology. (2019) 108:343–53. doi: 10.1159/000498887 30759445

[16] de Mestier L Walter T Evrard C de Boissieu P Hentic O Cros J . Temozolomide alone or combined with capecitabine for the treatment of advanced pancreatic neuroendocrine tumor. Neuroendocrinology. (2020) 110:83–91. doi: 10.1159/000500862 31071715 PMC6979423

[17] Dilz LM Denecke T Steffen IG Prasad V von Weikersthal LF Pape UF . Streptozocin/5-fluorouracil chemotherapy is associated with durable response in patients with advanced pancreatic neuroendocrine tumours. Eur J Cancer. (2015) 51:1253–62. doi: 10.1016/j.ejca.2015.04.005 25935542

[18] Ducreux M Dahan L Smith D O'Toole D Lepère C Dromain C . Bevacizumab combined with 5-FU/streptozocin in patients with progressive metastatic well-differentiated pancreatic endocrine tumours (BETTER trial): a phase II non-randomised trial. Eur J Cancer. (2014) 50:3098–106. doi: 10.1016/j.ejca.2014.10.002 25454412

[19] Dussol AS Joly MO Vercherat C Forestier J Hervieu V Scoazec JY . Gemcitabine and oxaliplatin or alkylating agents for neuroendocrine tumors: comparison of efficacy and search for predictive factors guiding treatment choice. Cancer. (2015) 121:3428–34. doi: 10.1002/cncr.29517 26058464

[20] Faure M Niccoli P Autret A Cavaglione G Mineur L Raoul JL . Systemic chemotherapy with FOLFOX in metastatic grade 1/2 neuroendocrine cancer. Mol Clin Oncol. (2017) 6:44–8. doi: 10.3892/mco.2016.1097 28123727 PMC5245060

[21] Ferrarotto R Testa L Riechelmann RP Sahade M Siqueira LT Costa FP . Combination of capecitabine and oxaliplatin is an effective treatment option for advanced neuroendocrine tumors. Rare Tumors. (2013) 5:e35. doi: 10.4081/rt.2013.e35 24179647 PMC3804810

[22] Firdaus I Shih KC Zakari A Lang EZ McCleod M Alguire KB . Bevacizumab, pertuzumab, and Sandostatin for patients with advanced neuroendocrine cancers [abstract. J Clin Oncol. (2012) 30:4127. doi: 10.1200/JCO.2012.30.15_suppl.412742697810

[23] Frilling A Modlin IM Kidd M Russell C Breitenstein S Salem R . Recommendations for management of patients with neuroendocrine liver metastases. Lancet Oncol. (2014) 15:e8–e21. doi: 10.1016/S1470-2045(13)70362-0 24384494

[24] Girot P Baudin E Senellart H Bouarioua N Hentic O Guimbaud R . Oxaliplatin and 5-fluorouracil in advanced well-differentiated digestive neuroendocrine tumors: a multicenter national retrospective study from the French Group of Endocrine Tumors. Neuroendocrinology. (2022) 112:537–46. doi: 10.1159/000518650 34348346

[25] Keppler-Noreuil KM Parker VE Darling TN Martinez-Agosto JA . Somatic overgrowth disorders of the PI3K/AKT/mTOR pathway and therapeutic strategies. Am J Med Genet C Semin Med Genet. (2016) 172:402–21. doi: 10.1002/ajmg.c.31531 27860216 PMC5592089

[26] Koumarianou A Kaltsas G Kulke MH Oberg K Strosberg JR Spada F . Temozolomide in advanced neuroendocrine neoplasms: pharmacological and clinical aspects. Neuroendocrinology. (2015) 101:274–88. doi: 10.1159/000430816 25924937

[27] Kunz PL Balise RR Fehrenbacher L Pan M Venook AP Fisher GA . Oxaliplatin-fluoropyrimidine chemotherapy plus bevacizumab in advanced neuroendocrine tumors: an analysis of two phase II trials. Pancreas. (2016) 45:1394–400. doi: 10.1097/MPA.0000000000000659 27171514

[28] Kunz PL Graham NT Catalano PJ Nimeiri HS Fisher GA Longacre TA . Randomized study of temozolomide or temozolomide and capecitabine in patients with advanced pancreatic neuroendocrine tumors (ECOG-ACRIN E2211). J Clin Oncol. (2023) 41:1359–69. doi: 10.1200/JCO.22.01013 36260828 PMC9995105

[29] Lacombe C Perrier M Hentic O Brixi H De Rycke O Cros J . FOLFOX-bevacizumab chemotherapy in patients with metastatic neuroendocrine tumors. J Neuroendocrinol. (2023) 35:e13227. doi: 10.1111/jne.13227 36599808 PMC10078167

[30] Lin CP Traets JJH Vredevoogd DW Visser NL Peeper DS . TSC2 regulates tumor susceptibility to TRAIL-mediated T-cell killing by orchestrating mTOR signaling. EMBO J. (2023) 42:e111614. doi: 10.15252/embj.2022111614 36715448 PMC9975943

[31] Maratta MG Sparagna I Occhipinti D Roca L Sgambato M Raia S . Upfront oxaliplatin-fluoropyrimidine chemotherapy and somatostatin analogues in advanced well-differentiated gastro-entero-pancreatic neuroendocrine tumors. Cancers (Basel). (2025) 17:1561. doi: 10.3390/cancers17091561 40361487 PMC12071586

[32] Merola E Dal Buono A Denecke T Arsenic R Pape UF Jann H . Efficacy and toxicity of 5-fluorouracil-oxaliplatin in gastroenteropancreatic neuroendocrine neoplasms. Pancreas. (2020) 49:912–7. doi: 10.1097/MPA.0000000000001593 32658073

[33] Mitry E Baudin E Ducreux M Sabourin JC Rufié P Aparicio T . Treatment of poorly differentiated neuroendocrine tumours with etoposide and cisplatin. Br J Cancer. (1999) 81:1351–5. doi: 10.1038/sj.bjc.6690325 10604732 PMC2362979

[34] Mitry E Walter T Baudin E Kurtz JE Ruszniewski P Dominguez-Tinajero S . Bevacizumab plus capecitabine in patients with progressive advanced well-differentiated neuroendocrine tumors of the gastrointestinal tract (BETTER trial): a phase II non-randomised trial. Eur J Cancer. (2014) 50:3107–15. doi: 10.1016/j.ejca.2014.10.001 25454413

[35] Moertel CG Kvols LK O'Connell MJ Rubin J . Treatment of neuroendocrine carcinomas with combined etoposide and cisplatin: evidence of major therapeutic activity in the anaplastic variants of these neoplasms. Cancer. (1991) 68:227–32. doi: 10.1002/1097-0142(19910715)68:2<227::AID-CNCR2820680202>3.0.CO;2-I 1712661

[36] Oberg KE Reubi JC Kwekkeboom DJ Krenning EP . Role of somatostatins in gastroenteropancreatic neuroendocrine tumor development and therapy. Gastroenterology. (2010) 139:742–53.e1. doi: 10.1053/j.gastro.2010.07.002 20637207

[37] Pavel M O'Toole D Costa F Capdevila J Gross D Kianmanesh R . ENETS consensus guidelines update for the management of distant metastatic disease of intestinal, pancreatic, and bronchial neuroendocrine neoplasms and neuroendocrine neoplasms of unknown primary site. Neuroendocrinology. (2016) 103:172–85. doi: 10.1159/000443167 26731013

[38] Raymond E Dahan L Raoul JL Bang YJ Borbath I Lombard-Bohas C . Sunitinib malate for the treatment of pancreatic neuroendocrine tumors. N Engl J Med. (2011) 364:501–13. doi: 10.1056/NEJMoa1003825 21306237

[39] Sorbye H Kong G Grozinsky-Glasberg S Strosberg J . PRRT in high-grade digestive neuroendocrine neoplasms (NET G3 and NEC). J Neuroendocrinol. (2025) 37:e13443. doi: 10.1111/jne.13443 39243213 PMC11919471

[40] Spada F Antonuzzo L Marconcini R Radice D Antonuzzo A Ricci S . Oxaliplatin-based chemotherapy in advanced neuroendocrine tumors: clinical outcomes and preliminary correlation with biological factors. Neuroendocrinology. (2016) 103:806–14. doi: 10.1159/000444087 26789262

[41] Strosberg JR Fine RL Choi J Nasir A Coppola D Chen DT . First-line chemotherapy with capecitabine and temozolomide in patients with metastatic pancreatic endocrine carcinomas. Cancer. (2011) 117:268–75. doi: 10.1002/cncr.25425 20824724 PMC4665634

[42] Terris B Scoazec JY Rubbia L Bregeaud L Pepper MS Ruszniewski P . Expression of vascular endothelial growth factor in digestive neuroendocrine tumours. Histopathology. (1998) 32:133–8. doi: 10.1046/j.1365-2559.1998.00321.x 9543669

[43] Unal C Saglam S . Metronomic temozolomide and bevacizumab: a safe and effective frontier for treating metastatic neuroendocrine tumors. Cancers (Basel). (2023) 15:5688. doi: 10.3390/cancers15235688 38067391 PMC10705735

[44] Xu J Shen L Bai C Wang W Li J Yu X . Surufatinib in advanced pancreatic neuroendocrine tumours (SANET-p): a randomised, double-blind, placebo-controlled, phase 3 study. Lancet Oncol. (2020) 21:1489–99. doi: 10.1016/S1470-2045(20)30493-9 32966810

[45] Yao JC Shah MH Ito T Bohas CL Wolin EM Van Cutsem E . Everolimus for advanced pancreatic neuroendocrine tumors. N Engl J Med. (2011) 364:514–23. doi: 10.1056/NEJMoa1009290 21306238 PMC4208619

[46] Zhu M Costello BA Yin J Pettinger AM Strosberg JR Erlichman C . Phase II trial of bevacizumab monotherapy in pancreatic neuroendocrine tumors. Pancreas. (2021) 50:1435–9. doi: 10.1097/MPA.0000000000001950 35041344

