# Supplementary material for: Efficacy and safety of bevacizumab plus XELOX in patients with unresectable locally advanced or metastatic neuroendocrine tumors
Source: Front Endocrinol (Lausanne). 2026 Sep 30;17:1939938. doi: 10.3389/fendo.2026.1939938 (PMC13661408; doi:10.3389/fendo.2026.1939938)
Supplement: Supplementary file 1 [file Supplementaryfile1.docx]

**Supplementary Table 1. Baseline clinicopathological characteristics and best RECIST 1.1 response to bevacizumab plus XELOX in patients with neuroendocrine tumors.**

| **Characteristics** | CR+PR/total  **(ORR %)** | **SD+PD**  **N= 42 (%)** | **CR+PR**  **N= 30 (%)** | **Whole cohort**  **N= 72 (%)** | ***χ*^2^** | ***P-*value** |
| --- | --- | --- | --- | --- | --- | --- |
| Sex |  |  |  |  | 0.086 | 0.770 |
| Female | 11/25 (44.0) | 14 (33.3) | 11 (36.7) | 25 (34.7) |  |  |
| Male | 19/47 (40.4) | 28 (66.7) | 19 (63.3) | 47 (65.3) |  |  |
| Age, years |  |  |  |  | 0.014 | 0.905 |
| <60 | 14/33 (42.4) | 19 (45.2) | 14 (46.7) | 33 (45.8) |  |  |
| ≥60 | 16/39 (41.0) | 23 (54.8) | 16 (53.3) | 39 (54.2) |  |  |
| Family history |  |  |  |  | 1.607 | 0.205 |
| No | 25/64 (39.1) | 39 (92.9) | 25 (83.3) | 64 (88.9) |  |  |
| Yes | 5/8 (62.5) | 3 (7.1) | 5 (16.7) | 8 (11.1) |  |  |
| Smoking |  |  |  |  | 0.000 | 1.000 |
| No | 25/60 (41.7) | 35 (83.3) | 25 (83.3) | 60 (83.3) |  |  |
| Yes | 5/12 (41.7) | 7 (16.7) | 5 (16.7) | 12 (16.7) |  |  |
| Drinking |  |  |  |  | 0.294 | 0.588 |
| No | 27/63 (42.9) | 36 (85.7) | 27 (90.0) | 63 (87.5) |  |  |
| Yes | 3/9 (33.3) | 6 (14.3) | 3 (10.0) | 9 (12.5) |  |  |
| Hypertension |  |  |  |  | 0.067 | 0.796 |
| No | 25/59 (42.4) | 34 (81.0) | 25 (83.3) | 59 (81.9) |  |  |
| Yes | 5/13 (38.5) | 8 (19.0) | 5 (16.7) | 13 (18.1) |  |  |
| Diabetes |  |  |  |  | 1.606 | 0.205 |
| No | 24/62 (38.7) | 38 (90.5) | 24 (80.0) | 62 (86.1) |  |  |
| Yes | 6/10 (60.0) | 4 (9.5) | 6 (20.0) | 10 (13.9) |  |  |
| Carcinoid syndrome |  |  |  |  | 0.089 | 0.765 |
| No | 29/69 (42.0) | 40 (95.2) | 29 (96.7) | 69 (95.8) |  |  |
| Yes | 1/3 (33.3) | 2 (4.8) | 1 (3.3) | 3 (4.2) |  |  |
| Primary tumour site |  |  |  |  | 12.815 | 0.077 |
| Pancreas | 14/26 (53.8) | 12 (28.6) | 14 (46.7) | 26 (36.1) |  |  |
| Colorectum | 4/14 (28.6) | 10 (23.8) | 4 (13.3) | 14 (19.4) |  |  |
| Small intestine | 1/3 (33.3) | 2 (4.8) | 1 (3.3) | 3 (4.2) |  |  |
| Mediastinum | 6/10 (60.0) | 4 (9.5) | 6 (20.0) | 10 (13.9) |  |  |
| Lung | 3/9 (33.3) | 6 (14.3) | 3 (10.0) | 9 (12.5) |  |  |
| Liver | 0/3 (0.0) | 3 (7.1) | 0 (0.0) | 3 (4.2) |  |  |
| Stomach | 2/2 (100.0) | 0 (0.0) | 2 (6.7) | 2 (2.8) |  |  |
| Others | 0/5 (0.0) | 5 (11.9) | 0 (0.0) | 5 (6.9) |  |  |
| Primary tumour site |  |  |  |  | 2.484 | 0.115 |
| Pancreas | 14/26 (53.8) | 12 (28.6) | 14 (46.7) | 26 (36.1) |  |  |
| Non-pancreas | 16/46 (34.8) | 30 (71.4) | 16 (53.3) | 46 (63.9) |  |  |
| Primary tumour site |  |  |  |  | 0.294 | 0.588 |
| Lung | 3/9 (33.3) | 6 (14.3) | 3 (10.0) | 9 (12.5) |  |  |
| Non-lung | 27/63 (42.9) | 36 (85.7) | 27 (90.0) | 63 (87.5) |  |  |
| Radiotherapy |  |  |  |  | 2.256 | 0.133 |
| No | 27/59 (45.8) | 32 (76.2) | 27 (90.0) | 59 (81.9) |  |  |
| Yes | 3/13 (23.1) | 10 (23.8) | 3 (10.0) | 13 (18.1) |  |  |
| Interventional therapy |  |  |  |  | 1.710 | 0.191 |
| No | 21/44 (47.7) | 23 (54.8) | 21 (70.0) | 44 (61.1) |  |  |
| Yes | 9/28 (32.1) | 19 (45.2) | 9 (30.0) | 28 (38.9) |  |  |
| Immunotherapy |  |  |  |  | 1.600 | 0.206 |
| No | 28/63 (44.4) | 35 (83.3) | 28 (93.3) | 63 (87.5) |  |  |
| Yes | 2/9 (22.2) | 7 (16.7) | 2 (6.7) | 9 (12.5) |  |  |
| Neoadjuvant therapy |  |  |  |  | 0.743 | 0.389 |
| No | 27/67 (40.3) | 40 (95.2) | 27 (90.0) | 67 (93.1) |  |  |
| Yes | 3/5 (60.0) | 2 (4.8) | 3 (10.0) | 5 (6.9) |  |  |
| Surgery |  |  |  |  | 3.575 | 0.059 |
| No | 21/41 (51.2) | 20 (47.6) | 21 (70.0) | 41 (56.9) |  |  |
| Yes | 9/31 (29.0) | 22 (52.4) | 9 (30.0) | 31 (43.1) |  |  |
| Number of lines of systemic treatment |  |  |  |  | 1.694 | 0.792 |
| 1 | 5/10 (50.0) | 5 (11.9) | 5 (16.7) | 10 (13.9) |  |  |
| 2 | 6/11 (54.5) | 5 (11.9) | 6 (20.0) | 11 (15.3) |  |  |
| 3 | 6/17 (35.3) | 11 (26.2) | 6 (20.0) | 17 (23.6) |  |  |
| 4 | 7/20 (35.0) | 13 (31.0) | 7 (23.3) | 20 (27.8) |  |  |
| ≥5 | 6/14 (42.9) | 8 (19.0) | 6 (20.0) | 14 (19.4) |  |  |
| Number of treatment cycles |  |  |  |  | 11.565 | 0.001 |
| <8 | 4/26 (15.4) | 22 (52.4) | 4 (13.3) | 26 (36.1) |  |  |
| ≥8 | 26/46 (56.5) | 20 (47.6) | 26 (86.7) | 46 (63.9) |  |  |
| Number of metastatic organs |  |  |  |  | 1.929 | 0.381 |
| No distant metastasis | 2/4 (50.0) | 2 (4.8) | 2 (6.7) | 4 (5.6) |  |  |
| ≤2 organs | 23/50 (46.0) | 27 (64.3) | 23 (76.7) | 50 (69.4) |  |  |
| ≥3 organs | 5/18 (27.8) | 13 (31.0) | 5 (16.7) | 18 (25.0) |  |  |
| Bone metastasis |  |  |  |  | 1.234 | 0.267 |
| No | 21/45 (46.7) | 24 (57.1) | 21 (70.0) | 45 (62.5) |  |  |
| Yes | 9/27 (33.3) | 18 (42.9) | 9 (30.0) | 27 (37.5) |  |  |
| Brain metastasis |  |  |  |  | 0.059 | 0.808 |
| No | 29/70 (41.4) | 41 (97.6) | 29 (96.7) | 70 (97.2) |  |  |
| Yes | 1/2 (50.0) | 1 (2.4) | 1 (3.3) | 2 (2.8) |  |  |
| Liver metastasis |  |  |  |  | 0.432 | 0.511 |
| No | 10/21 (47.6) | 11 (26.2) | 10 (33.3) | 21 (29.2) |  |  |
| Yes | 20/51 (39.2) | 31 (73.8) | 20 (66.7) | 51 (70.8) |  |  |
| Lung metastasis |  |  |  |  | 0.372 | 0.542 |
| No | 24/55 (43.6) | 31 (73.8) | 24 (80.0) | 55 (76.4) |  |  |
| Yes | 6/17 (35.3) | 11 (26.2) | 6 (20.0) | 17 (23.6) |  |  |
| Tumor size, cm |  |  |  |  | 0.159 | 0.690 |
| ≤3.5 | 15/34 (44.1) | 19 (45.2) | 15 (50.0) | 34 (47.2) |  |  |
| >3.5 | 15/38 (39.5) | 23 (54.8) | 15 (50.0) | 38 (52.8) |  |  |
| Grade |  |  |  |  | 0.197 | 0.906 |
| G1 | 3/6 (50.0) | 3 (7.1) | 3 (10.0) | 6 (8.3) |  |  |
| G2 | 19/46 (41.3) | 27 (64.3) | 19 (63.3) | 46 (63.9) |  |  |
| G3 | 8/20 (40.0) | 12 (28.6) | 8 (26.7) | 20 (27.8) |  |  |
| AJCC T staging |  |  |  |  | 3.314 | 0.346 |
| T1 | 5/20 (25.0) | 15 (35.7) | 5 (16.7) | 20 (27.8) |  |  |
| T2 | 15/30 (50.0) | 15 (35.7) | 15 (50.0) | 30 (41.7) |  |  |
| T3 | 8/18 (44.4) | 10 (23.8) | 8 (26.7) | 18 (25.0) |  |  |
| T4 | 2/4 (50.0) | 2 (4.8) | 2 (6.7) | 4 (5.6) |  |  |
| AJCC N staging |  |  |  |  | 0.530 | 0.767 |
| N0 | 7/19 (36.8) | 12 (28.6) | 7 (23.3) | 19 (26.4) |  |  |
| N1 | 19/42 (45.2) | 23 (54.8) | 19 (63.3) | 42 (58.3) |  |  |
| N2 | 4/11 (36.4) | 7 (16.7) | 4 (13.3) | 11 (15.3) |  |  |
| AJCC M staging |  |  |  |  | 0.006 | 0.938 |
| M0 | 2/4 (50.0) | 2 (4.8) | 2 (6.7) | 4 (5.6) |  |  |
| M1 | 28/68 (41.2) | 40 (95.2) | 28 (93.3) | 68 (94.4) |  |  |
| Vascular invasion |  |  |  |  | 2.244 | 0.326 |
| No | 5/18 (27.8) | 13 (31.0) | 5 (16.7) | 18 (25.0) |  |  |
| Yes | 6/15 (40.0) | 9 (21.4) | 6 (20.0) | 15 (20.8) |  |  |
| Unknown | 19/39 (48.7) | 20 (47.6) | 19 (63.3) | 39 (54.2) |  |  |
| Nerve invasion |  |  |  |  | 2.003 | 0.367 |
| No | 7/23 (30.4) | 16 (38.1) | 7 (23.3) | 23 (31.9) |  |  |
| Yes | 4/10 (40.0) | 6 (14.3) | 4 (13.3) | 10 (13.9) |  |  |
| Unknown | 19/39 (48.7) | 20 (47.6) | 19 (63.3) | 39 (54.2) |  |  |
| Ki-67 level |  |  |  |  | 0.714 | 0.700 |
| <5% | 2/5 (40.0) | 3 (7.1) | 2 (6.7) | 5 (6.9) |  |  |
| 5−10% | 8/23 (34.8) | 15 (35.7) | 8 (26.7) | 23 (31.9) |  |  |
| >10% | 20/44 (45.5) | 24 (57.1) | 20 (66.7) | 44 (61.1) |  |  |
| MGMT status |  |  |  |  | 0.535 | 0.765 |
| MGMT 0 or 1+ | 11/23 (47.8) | 12 (28.6) | 11 (36.7) | 23 (31.9) |  |  |
| MGMT 2+ or 3+ | 15/39 (38.5) | 24 (57.1) | 15 (50.0) | 39 (54.2) |  |  |
| Unknown | 4/10 (40.0) | 6 (14.3) | 4 (13.3) | 10 (13.9) |  |  |
| SSTR2 status |  |  |  |  | 0.192 | 0.909 |
| SSTR2 0 or 1+ | 12/29 (41.4) | 17 (40.5) | 12 (40.0) | 29 (40.3) |  |  |
| SSTR2 2+ or 3+ | 15/37 (40.5) | 22 (52.4) | 15 (50.0) | 37 (51.4) |  |  |
| Unknown | 3/6 (50.0) | 3 (7.1) | 3 (10.0) | 6 (8.3) |  |  |
| RB1 status |  |  |  |  | 0.268 | 0.875 |
| RB1 0 or 1+ | 21/48 (43.8) | 27 (64.3) | 21 (70.0) | 48 (66.7) |  |  |
| RB1 2+or 3+ | 4/11 (36.4) | 7 (16.7) | 4 (13.3) | 11 (15.3) |  |  |
| Unknown | 5/13 (38.5) | 8 (19.0) | 5 (16.7) | 13 (18.1) |  |  |
| PD-L1 CPS |  |  |  |  | 1.401 | 0.496 |
| 0 | 19/45 (42.2) | 26 (61.9) | 19 (63.3) | 45 (62.5) |  |  |
| ≥1 | 8/16 (50.0) | 8 (19.0) | 8 (26.7) | 16 (22.2) |  |  |
| unknown | 3/11 (27.3) | 8 (19.0) | 3 (10.0) | 11 (15.3) |  |  |
| MSS/MSI |  |  |  |  | 3.440 | 0.179 |
| MSS | 24/57 (42.1) | 33 (78.6) | 24 (80.0) | 57 (79.2) |  |  |
| MSI | 2/2 (100.0) | 0 (0.0) | 2 (6.7) | 2 (2.8) |  |  |
| unknown | 4/13 (30.8) | 9 (21.4) | 4 (13.3) | 13 (18.1) |  |  |
| Gene mutation |  |  |  |  | 4.380 | 0.112 |
| No | 10/33 (30.3) | 22 (52.4) | 10 (33.3) | 33 (45.8) |  |  |
| Yes | 9/13 (69.2) | 5 (11.9) | 9 (30.0) | 13 (18.1) |  |  |
| unknown | 11/26 (42.3) | 15 (35.7) | 11 (36.7) | 26 (36.1) |  |  |
| Ca19-9 (U/mL) |  |  |  |  | 0.196 | 0.658 |
| ≤ 20.53 | 18/41 (43.9) | 23 (54.8) | 18 (60.0) | 41 (56.9) |  |  |
| > 20.53 | 12/31 (38.7) | 19 (45.2) | 12 (40.0) | 31 (43.1) |  |  |
| Ca72-4 (U/mL) |  |  |  |  | 0.027 | 0.870 |
| ≤ 2.47 | 18/44 (40.9) | 26 (61.9) | 18 (60.0) | 44 (61.1) |  |  |
| > 2.47 | 12/28 (42.9) | 16 (38.1) | 12 (40.0) | 28 (38.9) |  |  |
| AFP (ng/mL) |  |  |  |  | 1.012 | 0.315 |
| ≤ 4.09 | 15/41 (36.6) | 26 (61.9) | 15 (50.0) | 41 (56.9) |  |  |
| > 4.09 | 15/31 (48.4) | 16 (38.1) | 15 (50.0) | 31 (43.1) |  |  |
| NSE (ng/mL) |  |  |  |  | 0.014 | 0.905 |
| ≤ 20.54 | 16/39 (41.0) | 23 (54.8) | 16 (53.3) | 39 (54.2) |  |  |
| > 20.54 | 14/33 (42.4) | 19 (45.2) | 14 (46.7) | 33 (45.8) |  |  |
| CEA (ng/mL) |  |  |  |  | 0.573 | 0.449 |
| ≤ 2.70 | 17/37 (45.9) | 20 (47.6) | 17 (56.7) | 37 (51.4) |  |  |
| > 2.70 | 13/35 (37.1) | 22 (52.4) | 13 (43.3) | 35 (48.6) |  |  |
| ProGRP (ng/mL) |  |  |  |  | 0.002 | 0.964 |
| ≤ 287.72 | 22/53 (41.5) | 31 (73.8) | 22 (73.3) | 53 (73.6) |  |  |
| > 287.72 | 8/19 (42.1) | 11 (26.2) | 8 (26.7) | 19 (26.4) |  |  |

Abbreviations: SD, stable disease; PD, progressive disease; CR, complete response; PR, partial response; MGMT, O6-methylguanine-DNA methyltransferase; SSTR2, somatostatin receptor 2; PD-L1 CPS, programmed death-ligand 1 combined positive score; RB1, retinoblastoma 1; MSS, microsatellite stable; MSI, microsatellite instability; CA19-9, carbohydrate antigen 19-9; CA72-4, carbohydrate antigen 72-4; AFP, alpha-fetoprotein; NSE, neuron-specific enolase; CEA, carcinoembryonic antigen; ProGRP, pro-gastrin-releasing peptide; AJCC, American Joint Committee on Cancer.

**Supplementary Figure 1. Kaplan–Meier subgroup estimates of overall survival.**

**Supplementary Figure 2. Kaplan–Meier subgroup estimates of progression-free survival.**
